# The Institution Garden

**Published:** 1918-03-16

**Authors:** 


### THE INSTITUTION GARDEN.

### I.—What to Do and How to Do it.

ALL cottage hospitals, and all asylums with the exception of Bethlem and St. Luke's, have gardens attached to them, and the management of the garden is always a very important part of the administration of an asylum, but was never as important as it is now. It is of national importance that every rod of ground that can be utilised to grow food should be utilised to the best advantage—that is to say, to grow the maximum quantity of food. There are hundreds of books about gardening, but among them all there is not one that treats of gardening from this point of view. The amateur gardener may learn from books how to grow any particular flower or vegetable. The professional or market gardener may learn from books how to treat his ground with respect to tillage, manuring, and so forth, so as to secure the maximum yield from the crops he cultivates, but no book will teach either of them how to crop his ground so as to obtain from it the maximum quantity of food; and this is the burning question for the gardener at the present moment.

### MONEY VALUE AND FOOD VALUE.

It does not follow that the crop that gives the greatest return in money will give the greatest return in food. The globe artichoke is a profitable vegetable from the money point of view, for its succulent heads command a good price in the market, and it requires scarcely any cultivation, and is not subject to disease; but a single plant of the artichoke will occupy a yard of ground. It will produce, at the utmost, half-a-dozen heads; and these heads put together (two heads are better than one) will contain no more than a couple of ounces or so of food. The same square yard of ground planted with potatoes will yield 20 lb. of food, the sale of which may bring in but little more than the half-dozen heads of the artichoke, but the food value is a hundred and sixty times as great.

### WEIGHT AND FOOD VALUE.

Nor is the weight of food produced the only thing to be considered. The same plot of ground will yield twice as much weight in cabbages as in potatoes; but 100 lb. of cabbages contain only 6½ lb. of dry food, but the 50 lb. of potatoes that can be grown on the same plot will contain 50 or 100 per cent. more food. Peas are richer in food, weight for weight, than potatoes, but while the potaoes produced from a rod of ground will weigh 160 lbs. or more, the peas produced from the same space of ground will weigh less than a tenth of this quantity. But then again the peas contain, weight for weight, three times as much protein, the most valuable constituent in the food, as potatoes, so that the problem is a complicated one, and is still further complicated by the advisability, nay, the necessity, of avoiding monotony, and of producing relishes, which have little food value, but which render other foods palatable, and assist their digestion.

### KINDS OF CROPS.

### FOODS AND RELISHES—TOPS AND BOTTOMS.

For the purpose of the gardener who looks to grow the maximum quantity of food and to turn his ground to the best use for the production of food, vegetables may be divided into food crops and relishes, and the food crops may again be divided into tops and bottoms, or green crops and root crops. Some, such as celery, leeks, and chicory, occupy an intermediate position in that they naturally grow above ground, but the gardener "earths them up" in order to blanch them and remove a bitterness that they have when green; but with respect to food value they rank with green crops.

### PERMANENT AND TEMPORARY CROPS.

Some crops, such as rhubarb, sea-kale, asparagus, and Jerusalem artichokes, occupy the same plot of ground year after year. The space they occupy in the garden is already apportioned to them, and although none of them has a high food value, the gardener is not likely to break up the ground and devote it to some other crop. These may be left out of our present consideration. The question that every gardener is revolving in his mind at this time of year is, What proportion of my land ought I to devote to this crop, and what proportion to that, so as to produce at once the maximum of food, paying due regard to the different ingredients of food—protein carbohydrates, and fats—and to the production of a wholesome and agreeable variety. This is the matter in which he will obtain no guidance from books on gardening, and this is the matter in which we propose to assist him.

### ROOT CROPS—POTATOES.

By far the more profitable in respect of the amount of food they produce per rod of ground are the root crops, which include potatoes, parsnips, carrots, turnips, beetroot, both the red and the sugar varieties, and swedes. Jerusalem artichokes, which also are a root crop, occupy a permanent plot, for if grown in different plots in successive years they infest the whole garden, and form a most troublesome weed springing up among other crops. Of the root crops, the potato bears the palm. Parsnips, carrots, and other root crops may produce as much weight per rod of ground, but of their weight from 87 to 93 per cent. consists of water, while no less than 25 per cent. of the potato is solid food. From every point of view, therefore, the potato is the most profitable food crop that can be grown; but there is this to be said against its cultivation in the garden—that it is more cheaply grown by field cultivation than by garden cultivation. Still, at the present time we want as much arable as possible for our cereals, and every rod of garden that is devoted to potatoes saves a rod of arable for cereals. And potatoes have another advantage as a garden crop, in that they, especially the early varieties, do not occupy the ground for long. They can soon be cleared off and make room for other crops. Taking one consideration with another, it is evident that potatoes should be largely grown. Not less than 20 per cent. of the garden should be occupied by them, and even 25 per cent. will not be too much. Next in value to the potato comes the parsnip, with 13 per cent. of solid food, 1½ per cent. being protein. Parsnips should therefore be largely grown, and 10 per cent. of the garden ground may well be allotted to them. The other root crops, beet, turnips, carrots, and swedes, stand much on a level with respect to their food value, and 8 per cent. of the ground may be allotted to each of them, so that the root crops together may occupy 62 per cent., or, say, two-thirds of the area of the garden.

### GREEN CROPS—CABBAGES.

Of these the cabbage tribe give by far the greater weight per rod of ground, but the food value of this weight is very small, only about 6½ per cent. being solid,

**THE INSTITUTION GARDEN—(*continued*).**

and the rest water. Other disadvantages of the brassicas are that they are hungry crops, and take a great deal out of the ground, and they occupy the ground for a very long time, some of them for the best part of a year. Their value is chiefly in the salts and vitamines they contain; but a large proportion of the salts is dissolved out in the boiling and thrown away, and some of the vitamines are destroyed by boiling. On the whole, therefore, they are an unprofitable crop, and there is no doubt that much of the very large space they occupy in most gardens might be put to more useful purposes. Nevertheless, cabbages are not without value as food. They introduce a wholesome variety into the diet: some of the salts and some of the vitamines remain after boiling; and they satisfy the craving of the stomach for bulk. Concentrated foods are ill-digested, and a small bulk of food, even of very nutritious food, does not satisfy hunger as the same value of food distributed and diluted through a larger bulk will do. A certain amount of ground, therefore, say 12 or 13 per cent. in all, may be allotted to the brassicas. The kinds that should be preferred will be subsequently considered.

Peas and Beans.

Peas and beans give a very moderate yield in comparison with the ground they occupy. The actual weight of crop per rod of ground is very small indeed in comparison with that of cabbage or savoy, but for all that peas and beans constitute a very valuable crop, and are well worth cultivating, and this for several reasons. In the first place, though the crop is small in weight, it is rich in protein. It contains a much larger proportion of this valuable food constituent than any other garden crop, or, indeed, than almost any other vegetable product. In the second place, peas and beans do not exhaust the soil. On the contrary, in one respect, and this the most important, they actually enrich it; for though they are grown for their fruit, and fruit is rich in protein, that is, in nitrogen, they leave the ground actually richer in nitrogen instead of poorer. This is because their roots are the favourite nidus of a nitrifying bacillus, which has the power of fixing the nitrogen contained in the air that permeates the soil, and of converting it into a form in which it can be absorbed by the roots of plants. In the third place, they do not occupy the ground for long. They are transient crops. Whereas root crops, except the early horse carrots and the six-weeks and other early turnips, occupy the ground for the whole season, and brassicas may occupy it for eight or ten months, peas are over in from three to four months, broad beans for about the same period, and dwarf and runner beans for even less. Peas and beans are therefore profitable crops from every point of view except that of a heavy yield for the ground occupied, and they compensate for this by their brief occupation, and by leaving the ground free and unexhausted, and, moreover, deeply cultivated for a succeeding crop the same year. By all means, therefore, grow plenty of peas and beans. A distinction must be made between the broad bean, which is grown for the seed alone, a seed rich in protein, and the dwarf and runner beans of which the whole fruit is consumed, and is consumed unripe, so that the seed forms but a small proportion of the edible part. The pod, as distinguished from the seed, is not rich in protein, and ranks only with green vegetables; and therefore it would be wise to allow the seed to mature, and grow the crop for beans in the form of haricots. Eight or 10 per cent. of the ground, or even more, may well be allotted to peas and beans.

(*To be continued.*)

